# Supplementary material for: Laser-assisted microbial culturomics
Source: Nat Commun. 2025 Nov 26;16:10614. doi: 10.1038/s41467-025-66804-7 (PMC12661035; doi:10.1038/s41467-025-66804-7)
Supplement: Supplementary file 1 — Supplementary Information [file 41467_2025_66804_MOESM1_ESM.pdf]

Supplementary Information for "Laser-assisted microbial culturomics"

Taoran Qu[1,2,9], Lothar Koch[2,3,4], Rumjhum Mukherjee[1,2], Yilin Tu[1,2,9], Amy L. Seidel[1,2], Lisan D. Püttmann[1,2], Andreas Winkel[1,2], Ines Yang[1,2], Jasmin Grischke[1,2], Dejia Liu[2,5], Willem F. Wolkers[2,5,6], Sophie Kittler[6,7], Boris Chichkov[2,3,4], Meike Stiesch[1,2,8] & Szymon P. Szafrański[1,2,8,*]

[1]Department of Prosthetic Dentistry and Biomedical Materials Science, Hannover Medical School, Carl-Neuberg-Str.1, 30625 Hannover, Germany

[2]Lower Saxony Centre for Biomedical Engineering, Implant Research and Development (NIFE), Hannover, Germany

[3]Institute of Quantum Optics, Leibniz Universität Hannover, Hannover, Germany

[4]Cluster of Excellence Rebirth (EXC 62), Leibniz Universität Hannover, Hannover, Germany

[5]Unit for Reproductive Medicine, University of Veterinary Medicine Hannover, Hannover, Germany

[6]Center for Translational Studies, University of Veterinary Medicine Hannover, Hannover, Germany

[7]Institute for Food Quality and Food Safety, University of Veterinary Medicine Hannover, Hannover, Germany

[8]Cluster of Excellence RESIST (EXC 2155), Hannover Medical School, Hannover, Germany

[9]Current working address: Shanghai Stomatological Hospital & School of Stomatology, Shanghai Key Laboratory of Craniomaxillofacial Development and Diseases, Fudan University, Shanghai, P. R. China

* Correspondence to: Dr. Szymon P. Szafrański, Department of Prosthetic Dentistry and Biomedical Materials Science, Hannover Medical School Carl-Neuberg-Str.1 30625 Hannover, Germany; Szafranski.Szymon@mh-hannover.de; Tel: +49 511 532 1415

List of contents

Supplementary Note 3

List of isolates incorporated in the culture collection 3

Supplementary references 6

Supplementary figures with legends 7

Supplementary Figure 1. Development of laser-assisted bioprinting for microbial inks. 7

Supplementary Figure 2. Application of membranes for improved management of bioprints. 8

Supplementary Figure 3. Bioprinting of diverse microbial inks. 9

Supplementary Figure 4. Bioprints representing clinical samples 10

Supplementary Figure 5. Composition and diversity of bioprints arrays at amplicon sequence variant level 12

Supplementary Figure 6. Rarefaction curves 14

Supplementary Figure 7. Characterization of enzymatic activity of reference strains 15

Supplementary Figure 8. FT-IR profiles of bioprints analyzed with PCA and ANN. 16

Supplementary Figure 9. FT-IR profiles of bioprints analyzed with LDA and RF. 17

Supplementary Figure 10. Comparative genomics of two Colibacter massiliensis strains from distinct habitats. 19

Supplementary Note

List of isolates incorporated in the culture collection

A total of 249 characterized isolates were incorporated in our culture collections (**Supplementary Data 6**). 84 strains originated from healthy individual A. 87, 20 and 58 came from individuals M, N and O, respectively, who all had peri-implantitis at the time of sampling. The isolates obtained in this study represent 14 classes of microorganisms, including bacteria and fungi. The following classes were represented (in alphabetic order): *Actinomycetia* (formerly *Actinobacteria*), *Alphaproteobacteria*, *Bacilli*, *Bacteroidia*, *Betaproteobacteria*, *Clostridia*, *Coriobacteriia*, *Epsilonproteobacteria* (currently "*Campylobacteria*"), *Erysipelotrichia*, *Flavobacteriia*, *Fusobacteriia*, *Gammaproteobacteria*, *Negativicutes* and *Saccharomycetes*. A total of 124 species were isolated. 30 different growth media used. In the following paragraphs the isolates have been grouped by class, alphabetized and listed with the identification numbers. The patient from which they were isolated and the medium used for isolation are indicated in brackets. Patients with peri-implantitis are indicated with '*'. Isolates were also summarized in context of their fastidious nature, ecological role and clinical relevance (**Supplementary Data 8**). Fastidious organisms were identified using Bergey's [1] and included those known to be hard to culture clinically [2], require specialized conditions , or remain uncultured/unamed oral taxa listed in the eHOMD database [3]. Relationship between isolates and active members of biofilms in peri-implantitits is showed in **Fig. 6** and **Supplementary Data 7**.

The *Actinomycetia* class was represented by 37 strains: *Actinomyces oris* SPS_755 (A, MSPS_074), *Actinomyces oris* SPS_966 (N*, MSPS_029), *Actinomyces oris* SPS_967 (O*, MSPS_029), *Actinomyces* sp. SPS_16 (M*, MSPS_023), *Actinomyces* sp. HMT-180 SPS_512 (M*, MSPS_029), *Actinomyces* sp. HMT-171 SPS_519 (M*, MSPS_029), *Actinomyces* sp. SPS_664 (M*, MSPS_023), *Actinomyces* sp. HMT-169 SPS_690 (O*, MSPS_023), *Actinomyces* sp. HMT-893/170/171 SPS_715 (N*, MSPS_039), *Actinomyces* sp. HMT-171 SPS_818 (A, MSPS_094), *Actinomyces* sp. HMT-169 SPS_829 (A, MSPS_074), *Actinomyces* sp. SPS_831 (A, MSPS_096), *Actinomyces* sp. SPS_840 (A, MSPS_030), *Actinomyces* sp. SPS_844 (A, MSPS_034), *Actinomyces* sp. HMT-169 SPS_853 (A, MSPS_100), *Actinomyces* sp. SPS_968 (O*, MSPS_029), *Arachnia rubra* SPS_828 (A, MSPS_029), *Bifidobacterium dentium* SPS_506 (M*, MSPS_029), *Bifidobacterium dentium* SPS_681 (O*, MSPS_029), *Bifidobacterium dentium* SPS_693 (O*, MSPS_029), *Corynebacterium durum* SPS_861 (A, MSPS_034), *Corynebacterium matruchotii* SPS_37 (M*, MSPS_002), *Cutibacterium acnes* SPS_666 (M*, MSPS_028), *Rothia dentocariosa* SPS_704 (O*, MSPS_035), *Rothia dentocariosa* SPS_771 (A, MSPS_094), *Rothia dentocariosa* SPS_841 (A, MSPS_030), *Rothia dentocariosa* SPS_987 (N*, MSPS_029), *Scardovia wiggsiae* SPS_517 (M*, MSPS_029), *Scardovia wiggsiae* SPS_721 (N*, MSPS_030), *Scardovia wiggsiae* SPS_842 (A, MSPS_030), *Scardovia wiggsiae* SPS_880 (N*, MSPS_042A), *Schaalia lingnae* [Not Validly Published] SPS_845 (A, MSPS_034), *Schaalia odontolytica* SPS_988 (M*, MSPS_029), *Schaalia* sp. SPS_679 (O*, MSPS_029), *Schaalia* sp. SPS_716 (N*, MSPS_030), *Schaalia* sp. SPS_851 (A, MSPS_034) and *Schaalia* sp. SPS_989 (O*, MSPS_029B).

The *Alphaproteobacteria* class was represented by a single strain: *Rhizobiaceae* sp. SPS_811 (A, MSPS_154).

The *Bacilli* class was represented by 79 strains: *Gemella haemolysans* SPS_695 (O*, MSPS_029), *Gemella haemolysans* SPS_765 (A, MSPS_029), *Gemella morbillorum* SPS_20 (M*, MSPS_028), *Gemella morbillorum* SPS_766 (A, MSPS_029), *Gemella morbillorum* SPS_978 (O*, MSPS_074), *Granulicatella adiacens* SPS_684 (O*, MSPS_029), *Granulicatella adiacens* SPS_718 (N*, MSPS_028), *Granulicatella adiacens* SPS_757 (A, MSPS_074), *Granulicatella adiacens* SPS_839 (A, MSPS_030), *Lactobacillus casei* SPS_490 (M*, MSPS_002), *Lactobacillus casei*

SPS_696 (O*, MSPS_002), *Lactobacillus casei* SPS_719 (N*, MSPS_023), *Lactobacillus gasseri* SPS_488 (M*, MSPS_006), *Lactobacillus gasseri* SPS_697 (O*, MSPS_007), *Lactobacillus paracasei* SPS_492 (M*, MSPS_002), *Lactobacillus paracasei* SPS_698 (O*, MSPS_002), *Lactobacillus paracasei* SPS_720 (N*, MSPS_042A), *Lactobacillus salivarius* SPS_491 (M*, MSPS_002), *Lactobacillus salivarius* SPS_699 (O*, MSPS_022), *Lactococcus lactis* SPS_685 (O*, MSPS_029), *Lactococcus lactis* SPS_700 (O*, MSPS_022), *Staphylococcus capitis* SPS_643 (M*, MSPS_037), *Staphylococcus epidermidis* SPS_644 (M*, MSPS_037), *Staphylococcus epidermidis* SPS_645 (N*, MSPS_034), *Staphylococcus epidermidis* SPS_758 (A, MSPS_074), *Staphylococcus epidermidis* SPS_830 (A, MSPS_102), *Streptococcus anginosus* SPS_004 (M*, MSPS_007), *Streptococcus anginosus* SPS_500 (M*, MSPS_035), *Streptococcus anginosus* SPS_523 (M*, MSPS_029), *Streptococcus anginosus* SPS_687 (O*, MSPS_029), *Streptococcus anginosus* SPS_707 (O*, MSPS_007), *Streptococcus anginosus* SPS_858 (A, MSPS_028), *Streptococcus australis* SPS_708 (O*, MSPS_029), *Streptococcus constellatus* SPS_51 (M*, MSPS_028), *Streptococcus constellatus* SPS_669 (M*, MSPS_034), *Streptococcus cristatus* SPS_14 (M*, MSPS_010), *Streptococcus gordonii* SPS_007 (M*, MSPS_007), *Streptococcus gordonii* SPS_017 (M*, MSPS_028), *Streptococcus gordonii* SPS_505 (M*, MSPS_029), *Streptococcus gordonii* SPS_709 (O*, MSPS_029), *Streptococcus gordonii* SPS_859 (A, MSPS_028), *Streptococcus gordonii* SPS_885 (A, MSPS_029), *Streptococcus gordonii* SPS_886 (A, MSPS_029), *Streptococcus gordonii* SPS_993 (N*, MSPS_029), *Streptococcus infantis* clade 431 SPS_498 (M*, MSPS_035), *Streptococcus infantis* clade 431 SPS_710 (O*, MSPS_029), *Streptococcus intermedius* SPS_723 (N*, MSPS_028), *Streptococcus intermedius* SPS_759 (A, MSPS_074), *Streptococcus intermedius* SPS_856 (A, MSPS_028), *Streptococcus lactarius* SPS_863 (A, MSPS_094), *Streptococcus mutans* SPS_499 (M*, MSPS_035), *Streptococcus mutans* SPS_857 (A, MSPS_028), *Streptococcus oralis* subsp. *tigurinus* clade 070 SPS_688 (O*, MSPS_029), *Streptococcus oralis* subsp. *tigurinus* clade 070 SPS_711 (O*, MSPS_022), *Streptococcus oralis* subsp. *tigurinus* clade 071 SPS_865 (N*, MSPS_023), *Streptococcus parasanguinis* clade 411 SPS_497 (M*, MSPS_035), *Streptococcus parasanguinis* clade 411 SPS_772 (A, MSPS_102), *Streptococcus parasanguinis* clade 721 SPS_670 (M*, MSPS_034), *Streptococcus parasanguinis* clade 721 SPS_712 (O*, MSPS_007), *Streptococcus salivarius* SPS_773 (A, MSPS_102), *Streptococcus sanguinis* SPS_774 (A, MSPS_096), *Streptococcus sanguinis* SPS_814 (A, MSPS_154), *Streptococcus sanguinis* SPS_816 (A, MSPS_154), *Streptococcus sanguinis* SPS_823 (A, MSPS_154), *Streptococcus* sp. (the Anginosus group) SPS_494 (M*, MSPS_035), *Streptococcus* sp. (the Anginosus group) SPS_763 (A, MSPS_074), *Streptococcus* sp. (the Mitis group) SPS_005 (M*, MSPS_007), *Streptococcus* sp. (the Mitis group) SPS_489 (M*, MSPS_037), *Streptococcus* sp. (the Mitis group) SPS_671 (M*, MSPS_037), *Streptococcus* sp. (the Mitis group) SPS_672 (M*, MSPS_037), *Streptococcus* sp. (the Mitis group) SPS_754 (A, MSPS_074), *Streptococcus* sp. (the Mitis group) SPS_809 (A, MSPS_154), *Streptococcus* sp. (the Mitis group) SPS_819 (A, MSPS_094), *Streptococcus* sp. (the Mitis group) SPS_860 (A, MSPS_030), *Streptococcus* sp. (the Salivarius group) SPS_713 (O*, MSPS_007), *Streptococcus* sp. HMT-064 SPS_810 (A, MSPS_154), *Streptococcus thermophilus* SPS_495 (M*, MSPS_035), *S. thermophilus* SPS_496 (M*, MSPS_035) and *Streptococcus vestibularis* SPS_775 (A, MSPS_102).

The *Bacteroidia* class was represented by 35 strains: *Alloprevotella rava* SPS_46 (M*, MSPS_004), *Alloprevotella rava* SPS_779 (A, MSPS_004), *Alloprevotella rava* SPS_969 (M*, MSPS_004), *Alloprevotella tannerae* SPS_41 (M*, MSPS_004), *Alloprevotella tannerae* SPS_970 (O*, MSPS_074), *Prevotella heparinolytica* (previously *Bacteroides heparinolyticus*) SPS_692 (O*, MSPS_029), *Porphyromonas endodontalis* SPS_801 (A, MSPS_029), *Porphyromonas gingivalis* SPS_982 (O*, MSPS_029), *Segatella* (previously *Prevotella*) *baroniae* SPS_45 (M*, MSPS_002), *Segatella* (previously *Prevotella*) *buccae* SPS_21 (M*, MSPS_004), *Segatella buccae* SPS_521 (M*, MSPS_029), *Segatella buccae* SPS_576 (O*, MSPS_042A2), *Segatella buccae* SPS_703 (O*, MSPS_029), *Prevotella denticola* SPS_49 (M*, MSPS_028), *Prevotella denticola* SPS_520 (M*, MSPS_029), *Prevotella denticola* SPS_577 (O*, MSPS_042A2), *Prevotella denticola* SPS_767 (A, MSPS_029), *Prevotella intermedia* SPS_983 (O*, MSPS_074), *Segatella* (previously *Prevotella*) *maculosa* SPS_50 (M*, MSPS_029), *Prevotella melaninogenica* SPS_820

(A, MSPS_029), *Prevotella multiformis* SPS_42 (M*, MSPS_002), *Prevotella multiformis* SPS_514 (M*, MSPS_029), *Prevotella nigrescens* SPS_22 (M*, MSPS_004), *Prevotella nigrescens* SPS_503 (M*, MSPS_029), *Prevotella nigrescens* SPS_686 (O*, MSPS_029), *Prevotella nigrescens* SPS_984 (O*, MSPS_151), *Segatella* (previously *Prevotella*) *oris* SPS_985 (O*, MSPS_004), *Prevotella pallens* SPS_769 (A, MSPS_004), *Prevotella* sp. HMT-313 SPS_843 (A, MSPS_030), *Prevotella* sp. HMT-314 SPS_502 (M*, MSPS_029), *Hoylesella* (previously *Prevotella*) sp. loescheii/HMT-472 SPS_986 (O*, MSPS_074), *Prevotella* veroralis SPS_24 (M*, MSPS_004), *Prevotella* veroralis SPS_770 (A, MSPS_004), *Prevotella* veroralis SPS_827 (A, MSPS_029) and *Prevotella* veroralis SPS_854 (A, MSPS_029).

The *Betaproteobacteria* class was represented by 13 strains: *Eikenella corrodens* SPS_010 (M*, MSPS_010), *Eikenella corrodens* SPS_852 (A, MSPS_099), *Kingella oralis* SPS_752 (A, MSPS_074), *Kingella oralis* SPS_848 (A, MSPS_010), *Neisseria bacilliformis* SPS_980 (N*, MSPS_029), *Neisseria elongata* SPS_825 (A, MSPS_154), *Neisseria mucosa* SPS_001 (M*, MSPS_002), *Neisseria mucosa* SPS_762 (A, MSPS_074), *Neisseria mucosa* SPS_812 (A, MSPS_154), *Neisseria mucosa* SPS_813 (A, MSPS_154), *Neisseria mucosa* SPS_826 (A, MSPS_154), *Neisseria subflava* SPS_821 (A, MSPS_154) and *Neisseria subflava* SPS_849 (A, MSPS_010).

The *Clostridia* class was represented by 8 strains: *Lachnoanaerobaculum* sp. SPS_753 (A, MSPS_074), *Oribacterium parvum* SPS_850 (A, MSPS_029), *Parvimonas micra* SPS_667 (M*, MSPS_030), *Parvimonas micra* SPS_702 (O*, MSPS_029), *Peptostreptococcaceae* [XI][G-1] [*Eubacterium*] *infirmum* SPS_511 (M*, MSPS_028), *Peptostreptococcaceae* [XI][G-1] [*Eubacterium*] *infirmum* SPS_838 (A, MSPS_028), *Shuttleworthia satelles* SPS_504 (M*, MSPS_029) and *Shuttleworthia satelles* SPS_705 (O*, MSPS_029).

The *Coriobacteriia* class was represented by 10 strains: *Lancefieldella parvula* SPS_510 (M*, MSPS_040), *Lancefieldella parvula* SPS_680 (O*, MSPS_029), *Lancefieldella parvula* SPS_691 (O*, MSPS_029), *Lancefieldella parvula* SPS_862 (A, MSPS_040), *Lancefieldella rimae* SPS_507 (M*, MSPS_040), *Olsenella uli* SPS_48 (M*, MSPS_029), *Olsenella uli* SPS_701 (O*, MSPS_028), *Olsenella uli* SPS_981 (O*, MSPS_029), *Slackia exigua* SPS_992 (O*, MSPS_074) and *Slackia exigua* SPS_706 (O*, MSPS_029).

The *Epsilonproteobacteria* class was represented by 4 strains: *Campylobacter concisus* SPS_768 (A, MSPS_041), *Campylobacter gracilis* SPS_971 (O*, MSPS_074), *Campylobacter showae* SPS_760 (A, MSPS_074) and *Campylobacter showae/rectus* SPS_972 (O*, MSPS_074).

The *Erysipelotrichia* class was represented by 3 strains: *Bulleidia extructa* SPS_665 (M*, MSPS_030), *Solobacterium moorei* SPS_059 (M*, MSPS_010) and *Solobacterium moorei* SPS_522 (M*, MSPS_029).

The *Flavobacteria* class was represented by 7 strains: *Capnocytophaga leadbetteri* SPS_15 (M*, MSPS_002), *Capnocytophaga leadbetteri* SPS_836 (A, MSPS_028), *Capnocytophaga* sp. (HMT 864-related) SPS_516 (M*, MSPS_028), *Capnocytophaga* sp. (HMT 864-related) SPS_973 (O*, MSPS_074), *Capnocytophaga* sp. HMT-324/326 SPS_835 (A, MSPS_028), *Capnocytophaga* sp. HMT-336 SPS_47 (M*, MSPS_002) and *Capnocytophaga* sp. HMT-864 SPS_493 (M*, MSPS_021).

The *Fusobacteriia* class was represented by 16 strains: *Fusobacterium nucleatum* SPS_977 (O*, MSPS_010), *Fusobacterium nucleatum* subsp. *animalis* SPS_063 (M*, MSPS_010), *Fusobacterium nucleatum* subsp. *animalis* SPS_683 (O*, MSPS_029), *Fusobacterium nucleatum* subsp. *animalis* SPS_694 (O*, MSPS_029), *Fusobacterium nucleatum* subsp. *animalis* SPS_717 (N*, MSPS_010), *Fusobacterium nucleatum* subsp. *vincentii* SPS_023 (M*, MSPS_004), *Fusobacterium nucleatum* subsp. *vincentii* SPS_060 (M*, MSPS_010), *Fusobacterium* sp. SPS_064 (M*, MSPS_010), *Fusobacterium* sp. SPS_847 (A, MSPS_010), *Leptotrichia hofstadii* SPS_003 (M*, MSPS_002), *Leptotrichia* sp. (wadei-related)

SPS_002 (M*, MSPS_002), *Leptotrichi*a sp. HMT-223 SPS_979 (O*, MSPS_074), *Leptotrichia* sp. HMT-225/buccalis SPS_56 (M*, MSPS_010), *Leptotrichia* sp. HMT-417 SPS_846 (A, MSPS_010), *Leptotrichia* sp. HMT-498 SPS_29 (M*, MSPS_002) and *Leptotrichia wadei* SPS_28 (M*, MSPS_002).

The *Gammaproteobacteria* class was represented by 5 strains: *Aggregatibacter aphrophilus* SPS_761 (A, MSPS_074), *Enterobacter* sp. SPS_532 (N*, MSPS_042A), *Haemophilus parainfluenzae* SPS_756 (A, MSPS_074), *Klebsiella* sp. SPS_817 (A, MSPS_154) and *Pseudomonas fluorescens* SPS_833 (A, MSPS_094).

The *Negativicutes* class was represented by 29 strains: *Anaeroglobus geminatus* SPS_501 (M*, MSPS_010), *Colibacter massiliensis* (previously *Megasphaera* sp. HMT-123) SPS_974 (O*, MSPS_151) see [4], *Dialister invisus* SPS_975 (O*, MSPS_074), *Dialister micraerophilus* SPS_682 (O*, MSPS_042C), *Dialister pneumosintes* SPS_012 (M*, MSPS_010), *Dialister pneumosintes* SPS_515 (M*, MSPS_028), *Dialister/Mitsuokella* sp. SPS_976 (O*, MSPS_151), *Selenomonas* sp. SPS_25 (M*, MSPS_029), *Selenomonas* sp. (HMT 136-related) SPS_668 (M*, MSPS_039), *Selenomonas* sp. HMT-136 SPS_832 (A, MSPS_041), *Selenomonas* sp. HMT-149 SPS_991 (N*, MSPS_029), *Selenomonas* sp. HMT-149/136 SPS_722 (N*, MSPS_030), *Selenomonas* sp. HMT-149/136 SPS_837 (A, MSPS_028), *Selenomonas* sp. HMT-501 SPS_58 (M*, MSPS_010), *Veillonella atypica* SPS_509 (M*, MSPS_040), *Veillonella atypica* SPS_776 (A, MSPS_041), *Veillonella dispar* SPS_013 (M*, MSPS_010), *Veillonella dispar* SPS_518 (M*, MSPS_029), *Veillonella dispar* SPS_724 (N*, MSPS_010), *Veillonella dispar* SPS_777 (A, MSPS_041), *Veillonella parvula* SPS_513 (M*, MSPS_029), *Veillonella parvula* SPS_778 (A, MSPS_099), *Veillonella parvula* SPS_824 (A, MSPS_154), *Veillonella parvula* SPS_855 (A, MSPS_041), *Veillonella* sp. SPS_508 (M*, MSPS_040), *Veillonella* sp. SPS_689 (O*, MSPS_029), *Veillonella* sp. SPS_714 (O*, MSPS_010), *Veillonella* sp. SPS_815 (A, MSPS_154) and *Veillonella* sp. SPS_822 (A, MSPS_154).

The *Saccharomycetes* class was represented by 2 strains: [*Candida*] *glabrata* SPS_524 (M*, MSPS_036) and *Candida* sp. SPS_887 (N*, MSPS_036).

Supplementary references

1 *Bergey's Manual of Systematic Bacteriology*. 2nd edn, Vol. 3-5 (2005-2012).

2 DOWNES, J. *et al.* Characterisation of Eubacterium-like strains isolated from oral infections. *Journal of Medical Microbiology* **50**, 947-951, doi:https://doi.org/10.1099/0022-1317-50-11-947 (2001).

3 Escapa, I. F. *et al.* New Insights into Human Nostril Microbiome from the Expanded Human Oral Microbiome Database (eHOMD): a Resource for the Microbiome of the Human Aerodigestive Tract. *mSystems* **3**, 10.1128/msystems.00187-00118, doi:doi:10.1128/msystems.00187-18 (2018).

4 Anani, H. *et al.* Colibacter massiliensis gen. nov. sp. nov., a novel Gram-stain-positive anaerobic diplococcal bacterium, isolated from the human left colon. *Scientific reports* **9**, 17199, doi:10.1038/s41598-019-53791-1 (2019).

Supplementary figures with legends

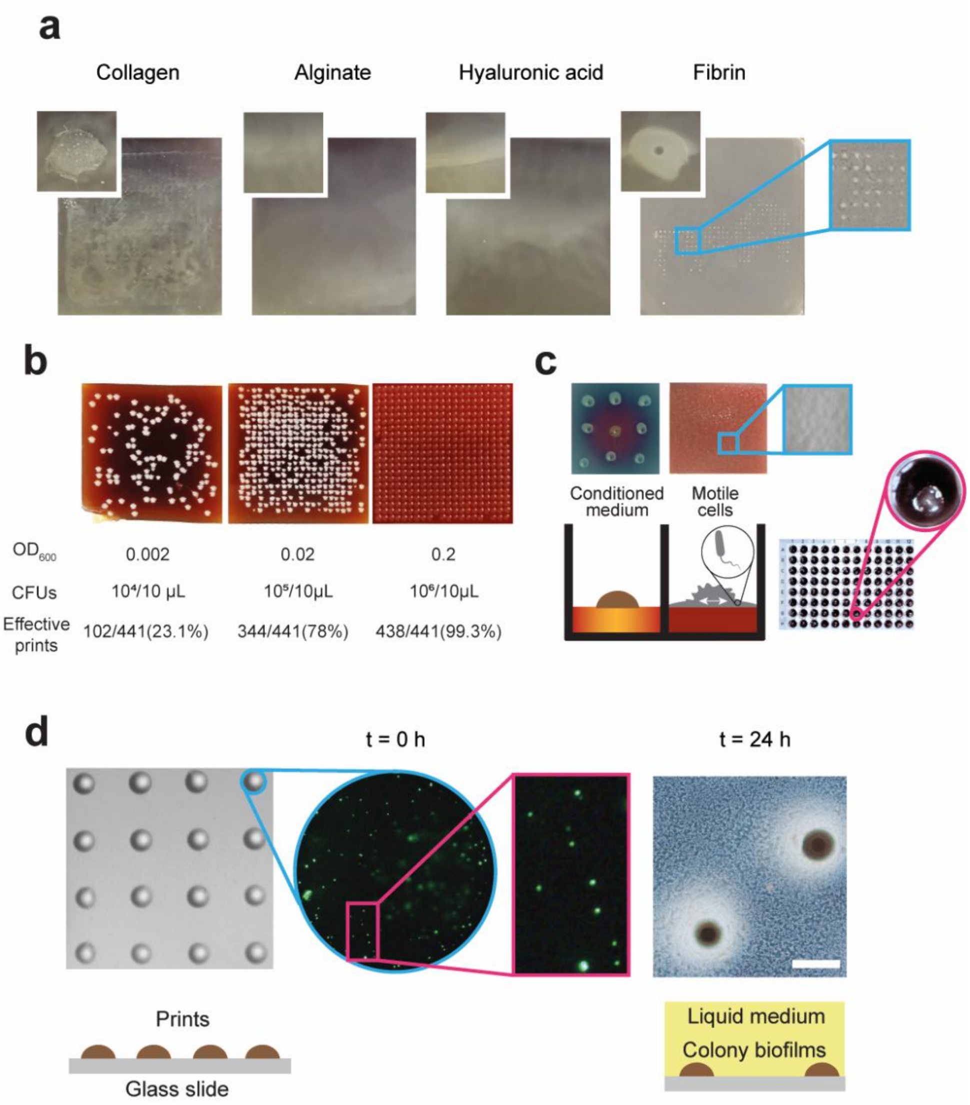

Supplementary Figure 1. Development of laser-assisted bioprinting for microbial inks.

a Bioprinting performance of inks consisting of cells of *Staphylococcus aureus* strain SPS_462, and either collagen, or alginate, or hyaluronic acid, or fibrin. Bioprinting was performed on a hydrogel-coated functionalized glass slide. Biofilm arrays were grown in liquid medium. Macroscopic pictures of bioprinted pattern (main) and bioink-dependent behavior (small) is shown. **b** The effect of cell concentration on printing efficiency. Bioprint size decrease with an increase in print density due to the higher cell number in a bioink. **c** Spatial separation of bioprints. Top left, conditioned medium with printed droplets, top right, motile biofilm after 24 h of growth, bottom right, medium-containing 96-well-plate after printing one droplet per well and incubation for 24 h. **d** For fibrin, which showed the highest quality of the bioprinting pattern, microscopic pictures show the bioprinting pattern and the location of viable cells (stained with Syto 9) in one fresh bioprint (t = 0 h) and developed colony biofilms (t = 24 h). Scale bar: 100 μm. Printing of model organisms using each co-printing technique was repeated at least twice, yielding consistent results.

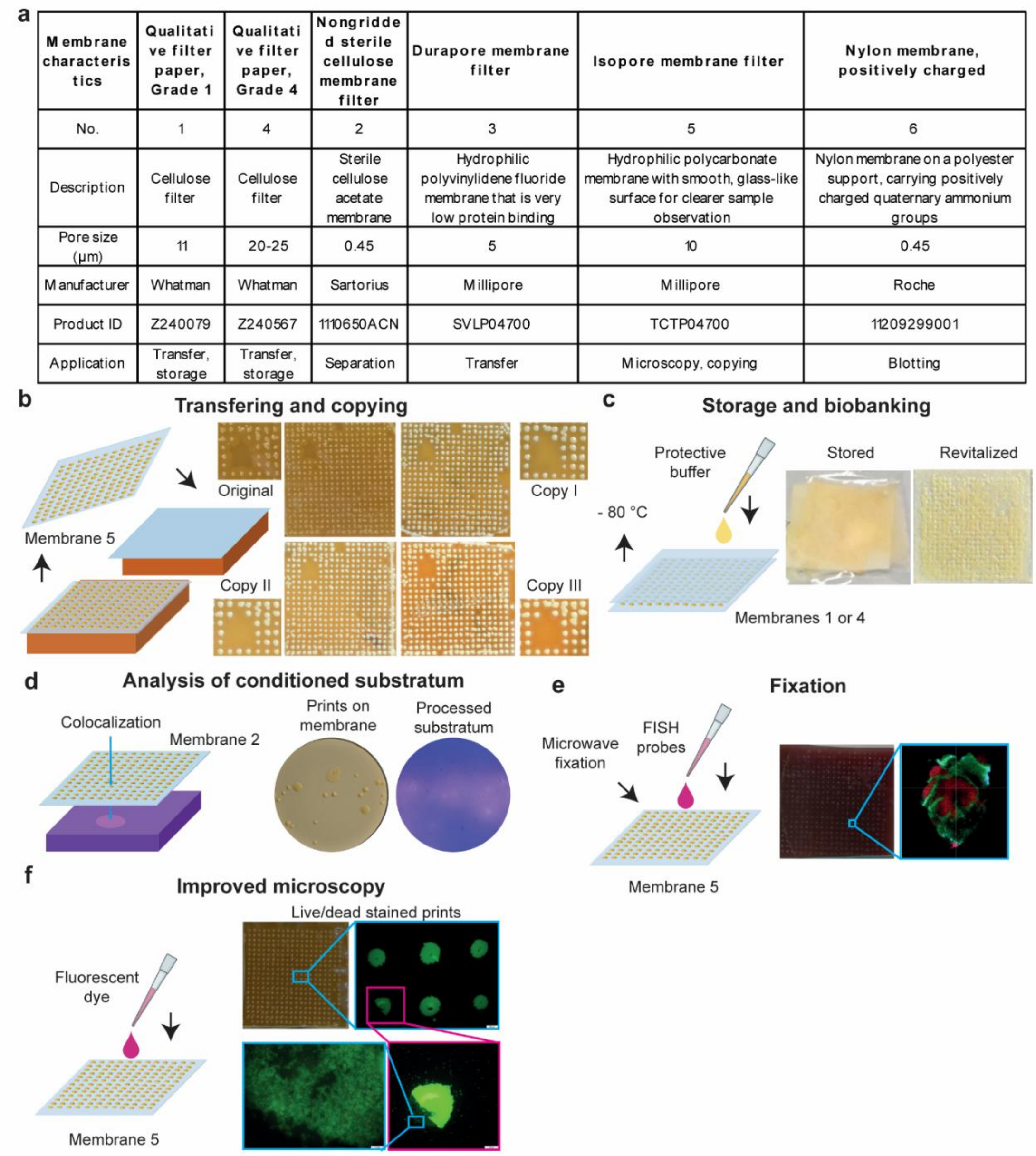

Membrane characteristics	Qualitative filter paper, Grade 1	Qualitative filter paper, Grade 4	Nongridded sterile cellulose membrane filter	Durapore membrane filter	Isopore membrane filter	Nylon membrane, positively charged
No.	1	4	2	3	5	6
Description	Cellulose filter	Cellulose filter	Sterile cellulose acetate membrane	Hydrophilic polyvinylidene fluoride membrane that is very low protein binding	Hydrophilic polycarbonate membrane with smooth, glass-like surface for clearer sample observation	Nylon membrane on a polyester support, carrying positively charged quaternary ammonium groups
Pore size (µm)	11	20-25	0.45	5	10	0.45
Manufacturer	Whatman	Whatman	Sartorius	Millipore	Millipore	Roche
Product ID	Z240079	Z240567	1110650ACN	SVLP04700	TCTP04700	11209299001
Application	Transfer, storage	Transfer, storage	Separation	Transfer	Microscopy, copying	Blotting

Supplementary Figure 2. Application of membranes for improved management of bioprints.

a Characteristics of membranes. **b** Transferring and copying of bioprinted biofilm arrays. Original arrays and their three copies. Photograph of the original array vertically mirrored to match copies. Top left area of bioprints was enlarged to better show the faithfulness of the copies. **c** Storage and revitalization of bioprints. Bioprints were stored between membranes in protective fluid at –80 °C. Subsequently, bioprints were retrieved from the biobank and copied onto fresh medium to revitalize them. **d** Removal of intact bioprints before treatment of medium for detection of enzymatic activity. Co-localization of bioprint and enzymatic activity allowed isolation of active strain. Plate is shown before and after membrane removal. **e** Bioprint fixation on membranes prior to probe hybridization. Visualization was performed with confocal scanning microscopy. **f** Improved microscopy for bioprints formed on membranes. Mono-species bioprints formed on membranes were stained with Syto 9 (green) and analyzed with confocal scanning microscopy. Each membrane-based experiment was repeated at least twice, consistently yielding reproducible results.

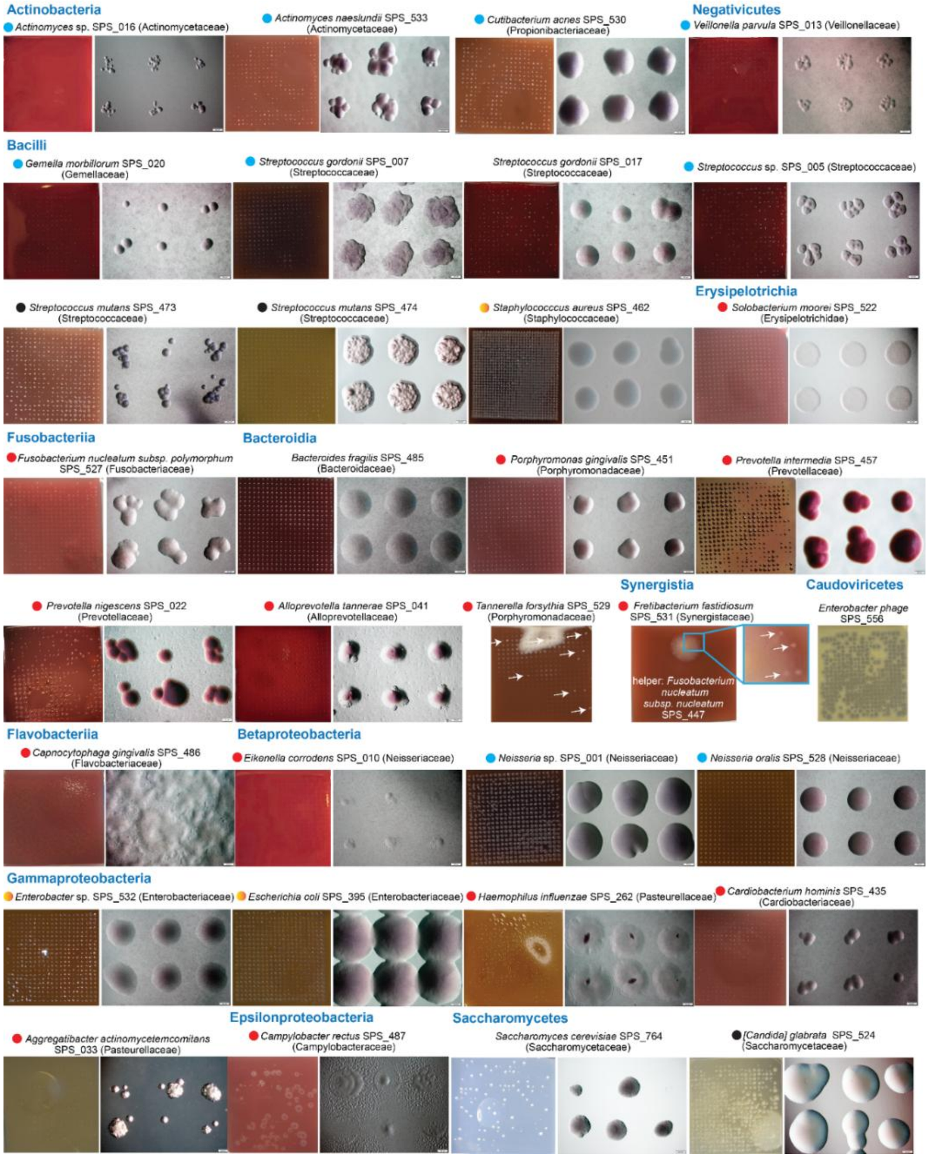

Supplementary Figure 3. Bioprinting of diverse microbial inks.

Microbial inks and resulting bioprint arrays. Macroscopic and microscopic pictures of bioprints representing diverse microbial species. Species are sorted by microbial class and taxonomy at the family level is listed in brackets for most of the taxa. Oral commensals, cariogenic species, periodontal pathogens and oral invaders are indicated with blue, black, red, and orange dot, respectively. Most strains represent either strict or facultative anaerobes and were bioprinted on the MSPS_029 medium, and cultured anaerobically (10% H_2, 10% CO_2, 80% N_2, 37°C). The fastidious strain SPS_529 was also cultured anaerobically but bioprinted on MSPS_074 flooded with N-acetylmuramic acid. SPS_531 required presence of helper strains SPS_447 for growth. SPS_001, SPS_005, SPS_033, SPS_395, SPS_404, SPS_424, SPS_435, SPS_462, SPS_528, SPS_532 were bioprinted on the MSPS_023 medium with or without sheep blood and cultured in 5% CO_2 at 37°C. The yeast SPS_764 was cultured in the same conditions but was bioprinted on the MSPS_036 medium. The bacteriophage SPS_556 was bioprinted on the semi-solid layer agar embedding cells of host strain SPS_532 (5% CO_2, 37°C). Bioprinting of the various strains was performed at least twice.

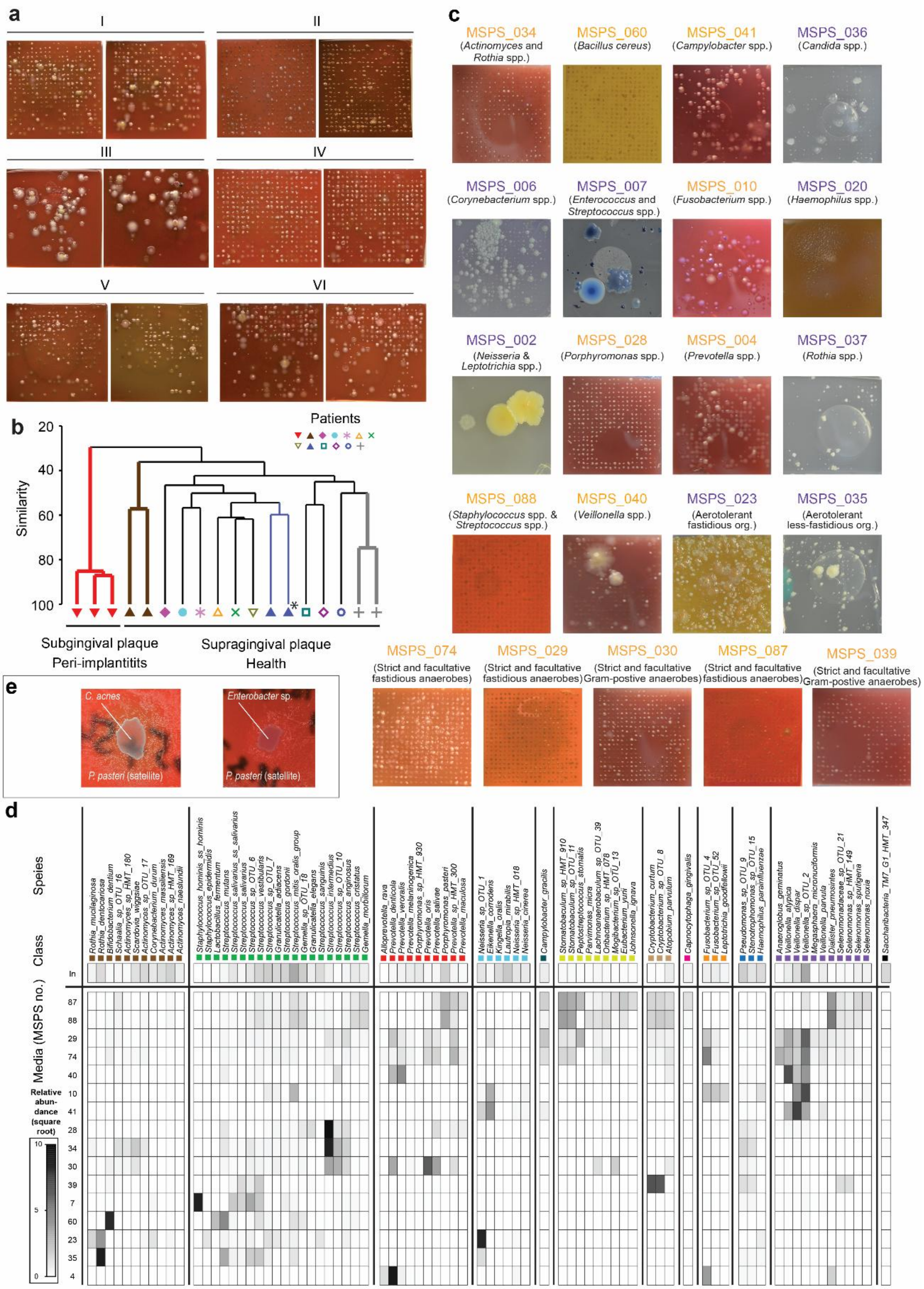

201

202 **Supplementary Figure 4.** Bioprints representing clinical samples

203 **a** Bioprinting on a single non-selective medium for fastidious anaerobes. Macroscopic pictures of bioprints representing six dental plaque samples,

204 each in two replicas. **b** Reproducibility of laser-assisted bioprinting of four samples (either in duplicate or triplicate) compared to nine other

samples assessed with 16S rRNA gene amplicons sequencing. 16S profiles are compared for 18 bioprint arrays of colony biofilms inoculated from 13 individuals. Two types of inocula were used: supragingival plaque from healthy individuals and submucosal plaques from peri-implantitits. Hierarchical cluster analysis was performed for Bray–Curtis similarity values that were calculated on standardized 4th root transformed abundances of reads grouped to species-level taxa. Transformation put a higher importance weight to low-abundant taxa. Communities from different patients are marked with different symbols. Colored lines link samples from the same individuals. * indicates that the patient was sampled on two different occasions. **c** Bioprinting on twenty-one media chosen to maximize recovery of oral microbial diversity. Macroscopic pictures of bioprints representing a single dental plaque sample. The target organisms are given in brackets. Medium identification number is colored by culture conditions: orange for anaerobic, violet for aerobic with 5% enriched CO_2. **d** Recovery of species for different media. Depicted are the most abundant species that reached at least 1% in original samples or in bioprints. Species were grouped by classes. Color scale, relative abundance on medium, square root of percent values. e Satellite growth of *Porphyromonas pasteri* around spots of the 'helper' strains: *Cutibacterium acnes* (left) and *Enterobacter* sp. (right). No growth was detected when *P. pasteri* was inoculated without a 'helper' strain. For additional details for b and d, refer to the **Source Data**.

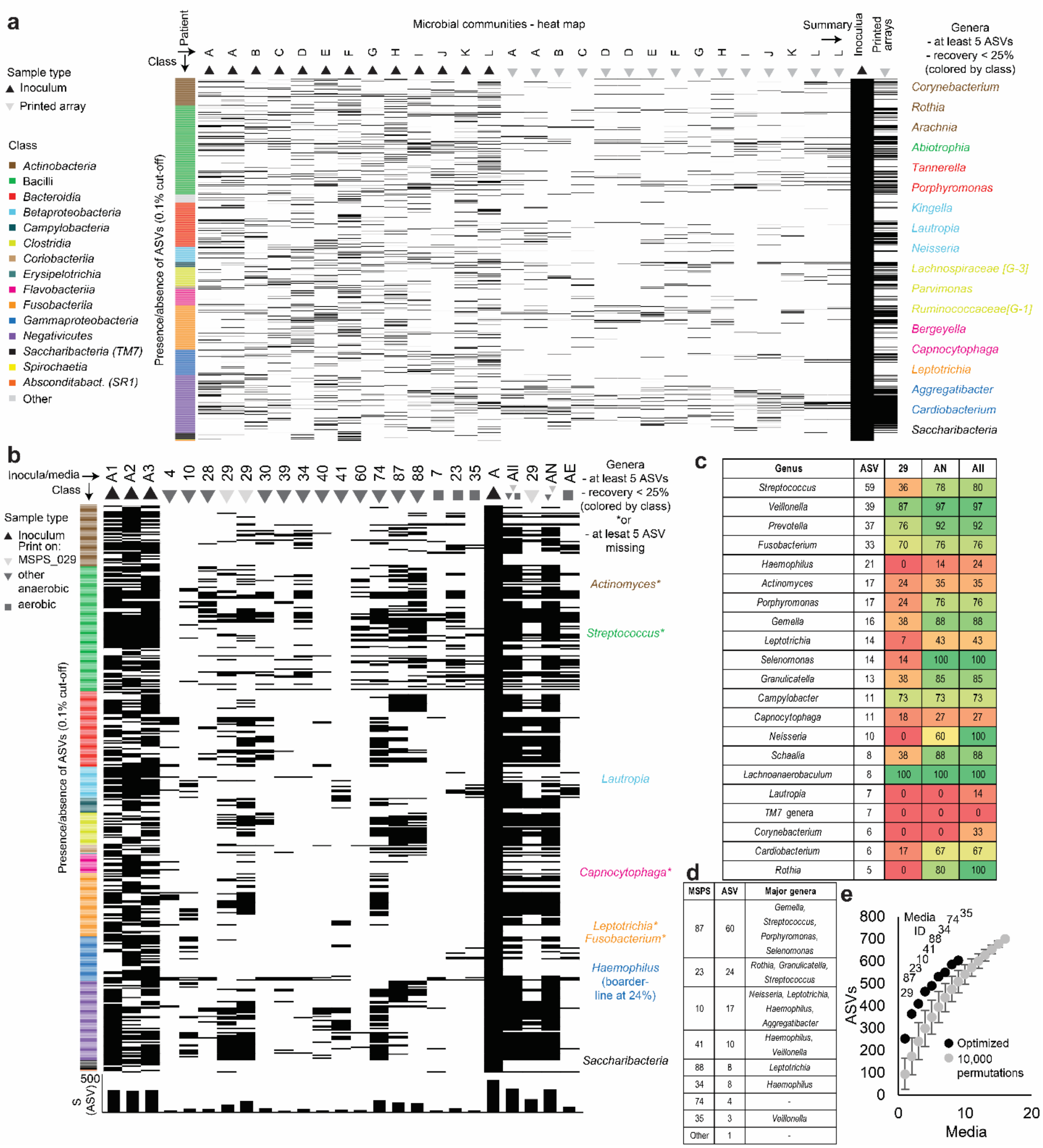

Supplementary Figure 5. Composition and diversity of bioprints arrays at amplicon sequence variant level

Composition and diversity of complex biofilm inocula and printed colony arrays as assessed at amplicon sequence (ASV) level. Data for prints on single robust medium and for culturomics are presented in a , and b - e, respectively. **a** Composition of colony arrays obtained on MSPS_029 medium (n = 12). The ASV and class levels are shown in the presence/absence heat map. ASVs were sorted by class. Labels depict selected genera from which individual ASVs present in the inocula were not recovered on medium MSPS_029. **b** Composition of colony arrays obtained by culturomics in comparison to the original samples. Data is shown like in ‘a’. Additionally, the ASVs’ richnes (equal to total ASVs number) of the colony arrays (S) is depicted under the graph. Mean values for inoculum, all culturomics, the MSPS_029 medium, all anaerobic media and all

aerobic media are shown on the right. Labels depict selected genera from which individual ASVs present in the inocula were not recovered by culturomics. Different symbols and numbers indicate the sample type or culture conditions, respectively. **c** Recovery of ASVs on medium MSPS_29 (29), all anaerobic media (AN) and culturomics, additionally including aerobic media (All). Genera represented by at least five ASVs identified in the inoculum were included in the analysis. **d** Stepwise evaluation of culture media conducted using UpSet plots to identify combinations that best complement each other in terms of ASV recovery. Beginning with MSPS_029 alone, we iteratively added the medium that contributed the highest number of unique ASVs at each step. This approach enabled us to rank the media based on their cumulative contribution and to highlight the distinct value of each medium within the overall collection. **e** ASV accumulation curves were generated to visualize the relationship between the optimized sequence of media combinations and those derived from 10,000 random permutations. These plots illustrate the efficiency of the optimized medium selection strategy in capturing ASV diversity compared to randomized orders. Error bars represent standard deviation (SD). For additional details on a - e, refer to the **Source Data**.

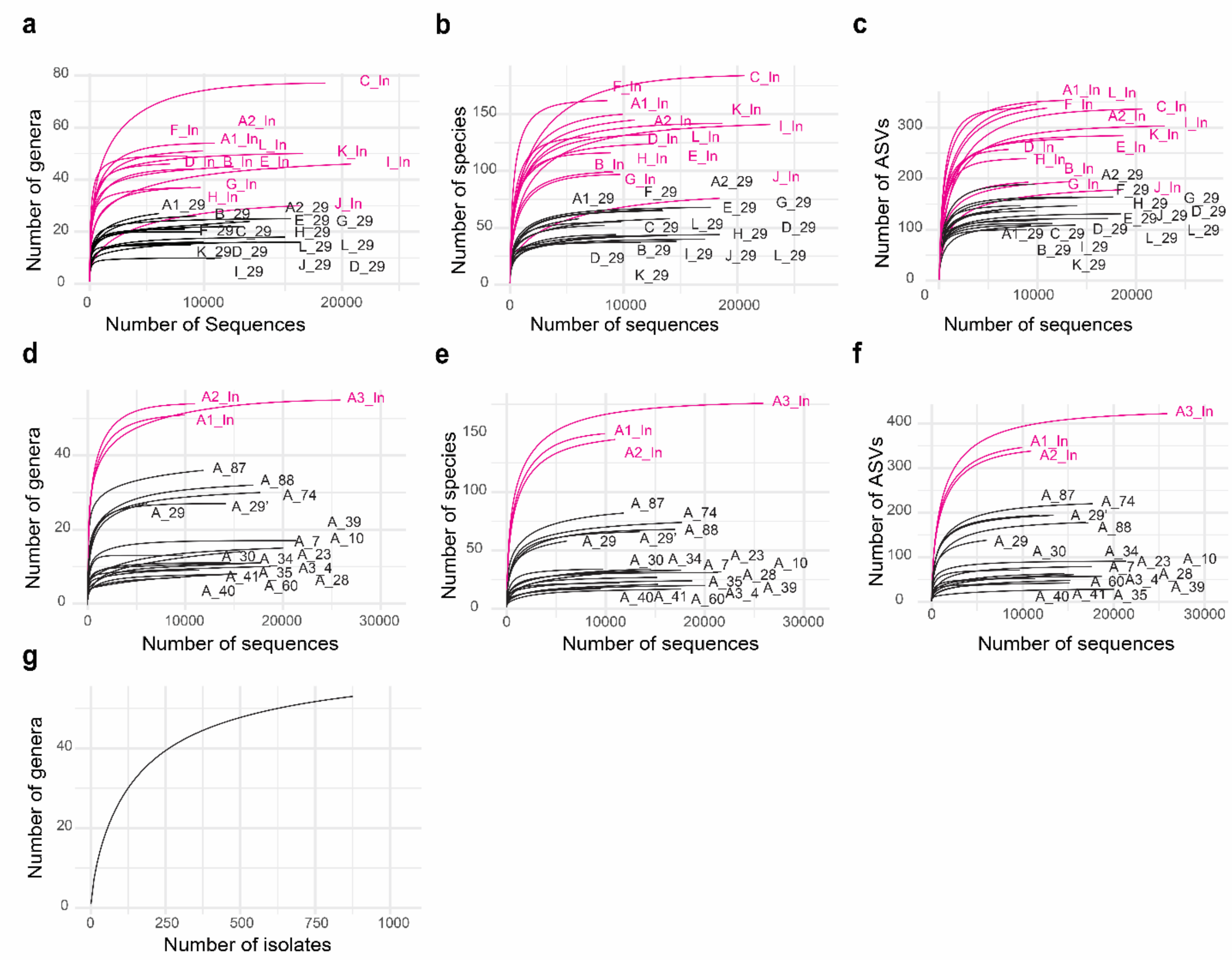

Supplementary Figure 6. Rarefaction curves

Rarefaction curves obtained for biofilm inocula (pink) compared to printed colony arrays on MSPS_029 (black). **a** Genus level. **b** Species level. **c** ASV level. Rarefaction curves obtained for biofilm inocula (pink) compared to printed colony arrays on culturomics media (black). **d** Genus level. **e** Species level. **f** ASV level. **g** A rarefaction curve obtained for isolates from 4 individuals generated at genus level. For additional details on a – g, refer to the **Source Data**.

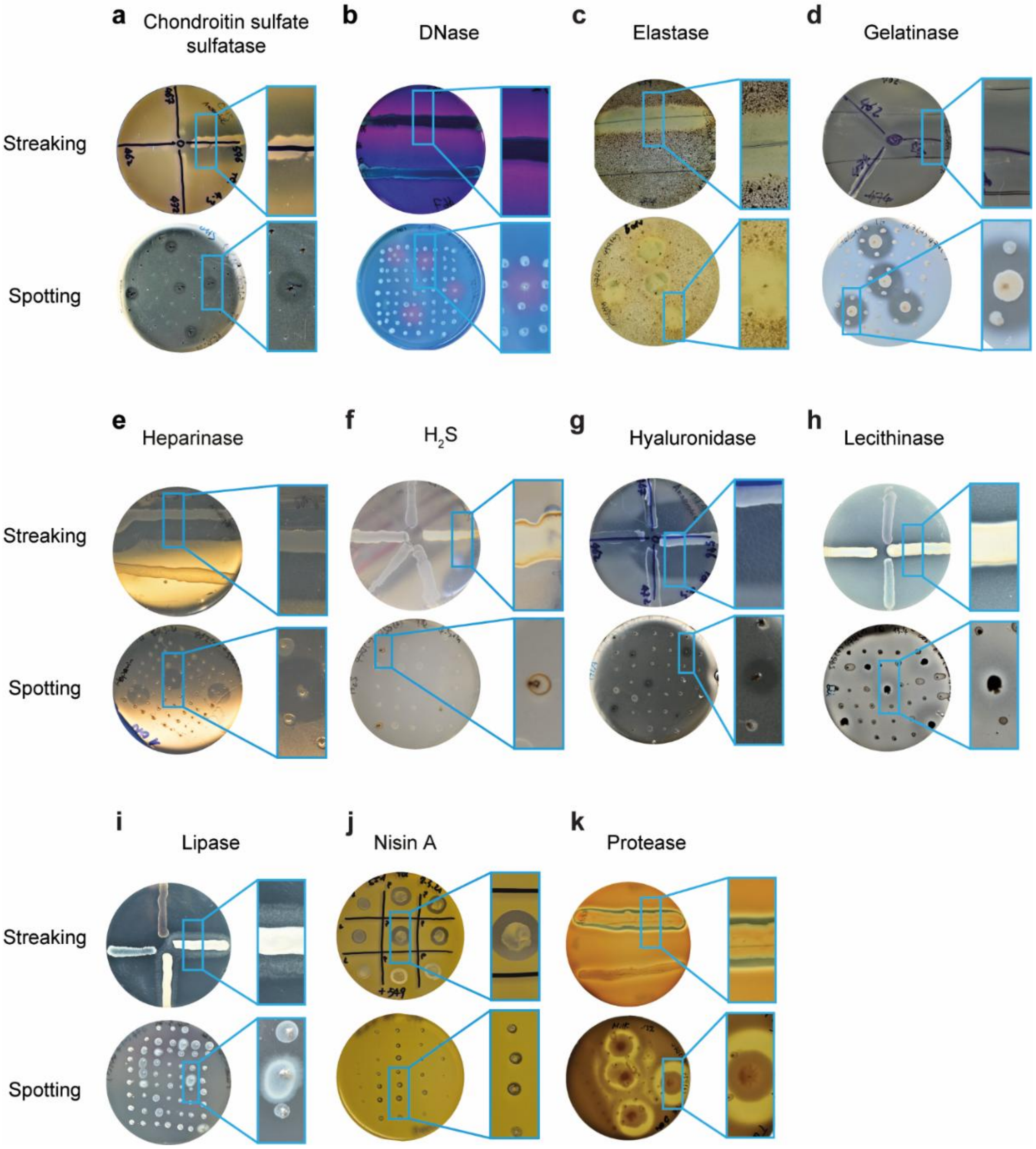

Supplementary Figure 7. Characterization of enzymatic activity of reference strains

Development and application of assays for detection of specific enzymatic activities in microbial streaks and spots on indicative solid media. Biochemical testing for detection of **a** chondroitin sulfate sulfatase, **b** DNAses, **c** elastase, **d** gelatinase, **e** heparinase, **f** hydrogen sulfide, **g** hyaluronidases, **h** lecithinase, **i** lipase, **j** bacteriocin, and **k** proteases (see SI). Manual streaks for reference strains (top). Manual spotting for mixed reference strains after adjusting testing conditions for strongly reduced biomass input (bottom). Areas showing positive signal are enlarged. Positive controls were *Cutibacterium acnes* SPS_546 (a, g, h, i), *Staphylococcus aureus* SPS_462 (b, g), *Pseudomonas aeruginosa* SPS_459 (c, h, i, k), *Bacillus* sp. SPS 554 (e), *Parvimonas micra* SPS_450 (f), *Porphyromonas gingivalis* SPS_451 (f), *Staphylococcus epidermidis* SPS_467 (d), and *Lactococcus lactis* subsp. *lactis* SPS_549, a bacteriocin nisin A producer, and *Micrococcus luteus* SPS_551, strain sensitive to nisin A, used for bacteriocin detection (j). Negative controls were *Streptococcus mutans* SPS_474 (a, c, d, f, g, h, i, k), *Staphylococcus epidermidis* SPS_467 (a, b, g), and *Staphylococcus aureus* SPS_462 (e, f, g).

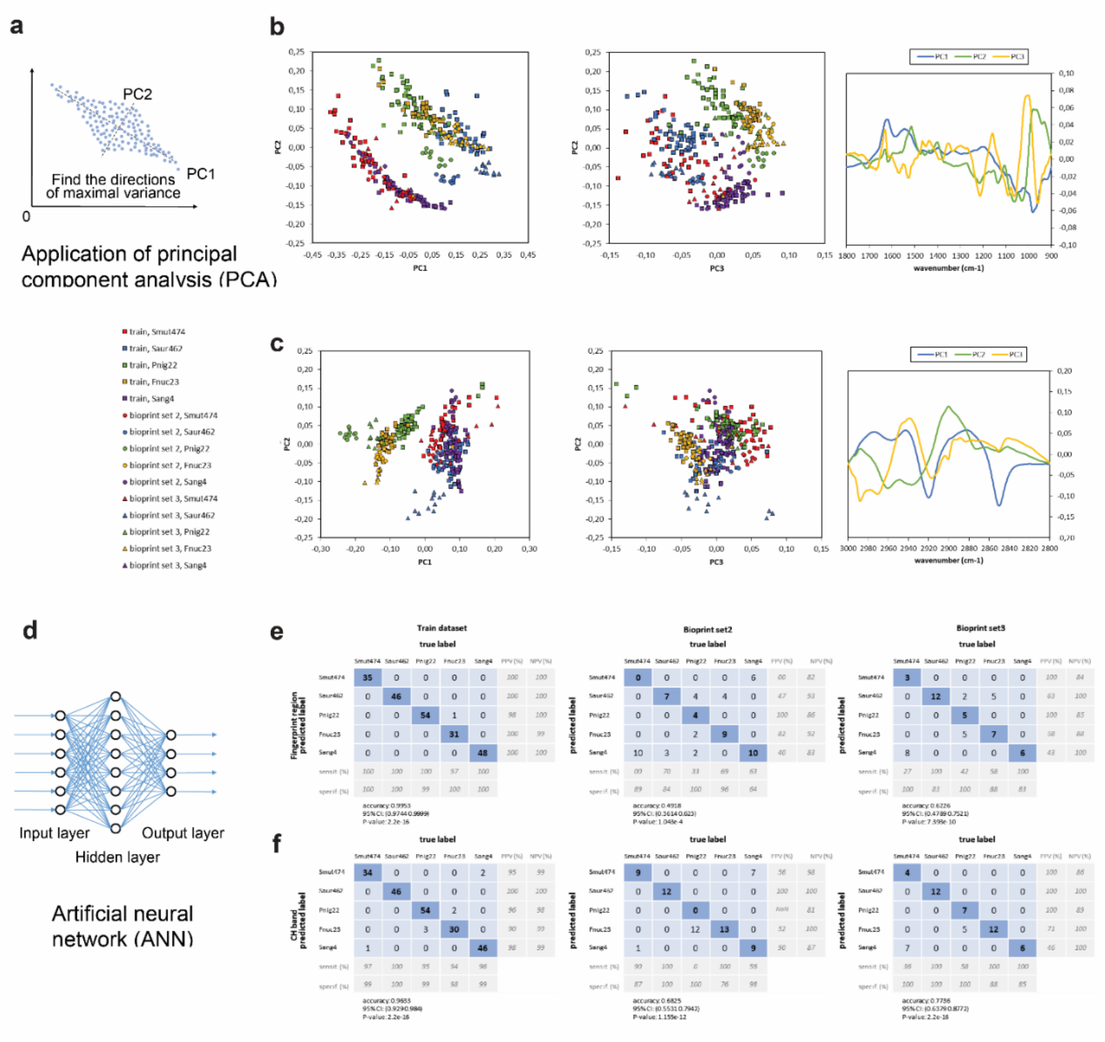

Supplementary Figure 8. FT-IR profiles of bioprints analyzed with PCA and ANN.

Principle component analysis (PCA) was used to create unconstrained ordinations. Artificial neural networks (ANN) were used to classify isolates originating from the bioprint arrays. **a** Schematic presentation of PCA. **b** PCA ordinations for fingerprint region of vector normalized FT-IR spectra generated for five strains (compare to Fig 5a – d). PC1 and PC2 axes are plotted left, PC2 and PC3 axes are plotted in the middle, while a loading plot of first three principal components in relationship to wavelength are plotted on the right. In the ordinations, different symbols indicate the training set and two independent test sets. Symbols were colored by taxonomy. **c** PCA ordinations for CH band of vector normalized FT-IR spectra generated for five strains. Data is presented like in “b”. **d** Schematic presentation of the artificial neural network (ANN). **e** ANN-predicted labels are plotted against true labels for fingerprint region of vector normalized FT-IR spectra generated for five strains. Data is presented separately for three data sets: training set (left), first test set (middle), and second test set (right). Matrix entries summarize assignments. Entries on the diagonal indicate matching labels (bold font on dark blue field). Misclassifications are listed in other cells (normal font on light blue field). Sensitivity and specificity are plotted for each row and column (light gray field). Finally, accuracy, 95% c.i., and p values are given below each matrix. **f** ANN-predicted labels are plotted against true labels for CH band of vector normalized FT-IR spectra generated for five strains. Data is presented like in “e”. In panels e and f, p-values were calculated using Pearson’s Chi-Square Test, and 95% confidence intervals were computed using the binom.test function. For additional details for b and c, refer to the **Source Data**.

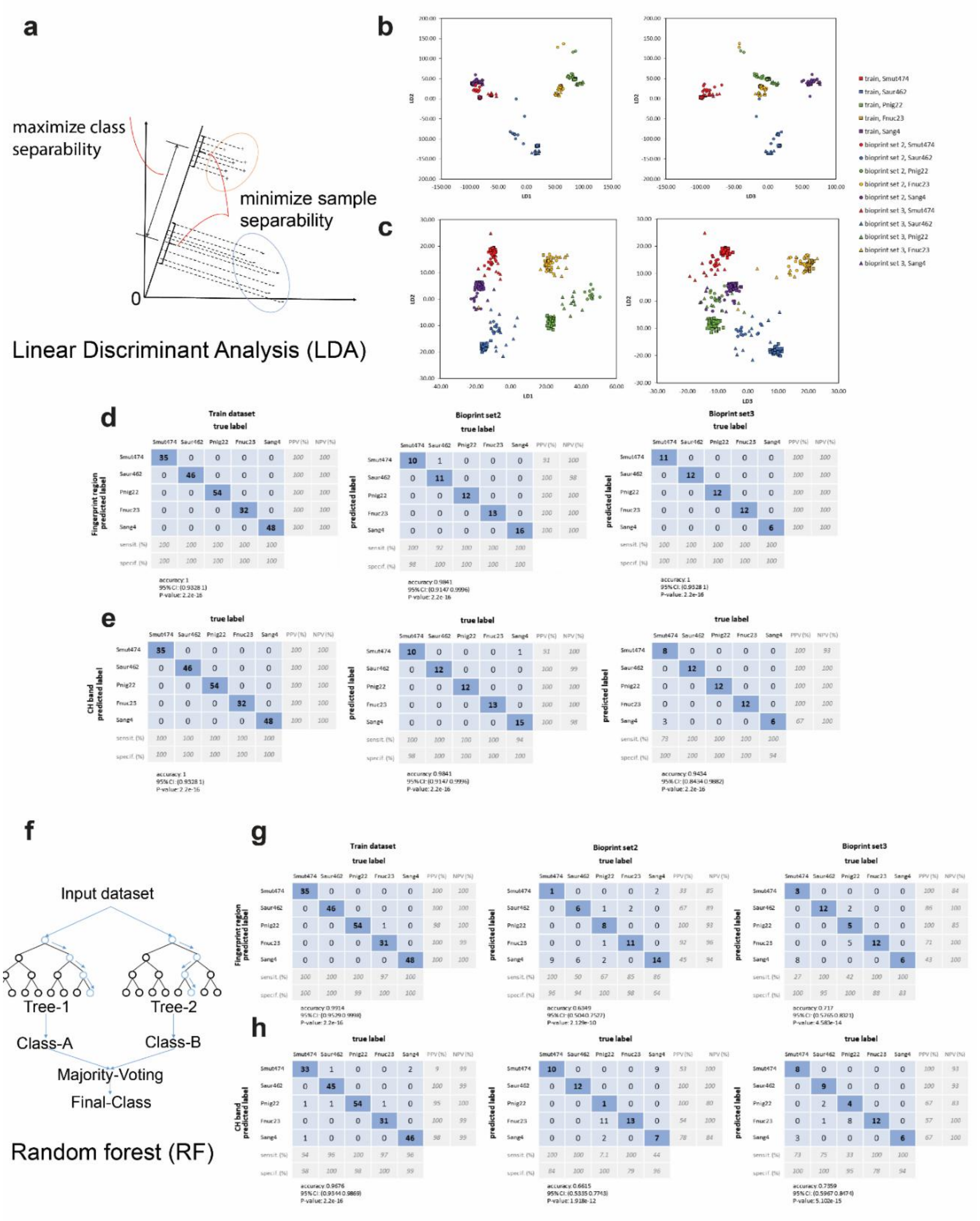

Supplementary Figure 9. FT-IR profiles of bioprints analyzed with LDA and RF.

Linear discriminant analysis (LDA) and random forest (RF) were used to classify bioprints based on same underlying raw data as presented in Fig. S5. **a** Schematic presentation of LDA. **b** Unconstrained LDA ordinations for fingerprint region of vector normalized FT-IR spectra fragments generated for five strains. LD1 and LD2 axes are plotted left while LD2 and LD3 axes are plotted right. Different symbols indicate training set and two independent test sets. Symbols were colored by taxonomy. **c** Unconstrained LDA ordinations for CH band of vector normalized FT-IR spectra fragments generated for five strains. Data is presented like in “b”. **d** LDA-predicted labels are plotted against true labels for fingerprint regions of

vector normalized FT-IR spectra generated for five strains. Data is presented separately for three data sets: training set (left), first test set (middle), and second test set (right). Matrix entries summarize assignments. Entries on diagonal indicate matching labels (bold font on dark blue field). Misclassifications are listed in other cells (normal font on light blue field). Sensitivity and specificity are plotted for each row and column (light gray field). Finally, accuracy, 95% c.i., and p values are given below each matrix. **e** LDA-predicted labels are plotted against true labels for CH band of vector normalized FT-IR spectra generated for five strains. Data is presented like in "d". **f** Schematic presentation of RF. **g** RF-predicted labels are plotted against true labels for fingerprint regions of vector normalized FT-IR spectra generated for five strains. Data is presented like in "d". **e** RF-predicted labels are plotted against true labels for CH band of vector normalized FT-IR spectra generated for five strains. Data is presented like in "d". In panels d, e, g and h, p-values were calculated using Pearson's Chi-Square Test, and 95% confidence intervals were computed using the binom.test function. For additional details on b and c, refer to the **Source Data**.

a

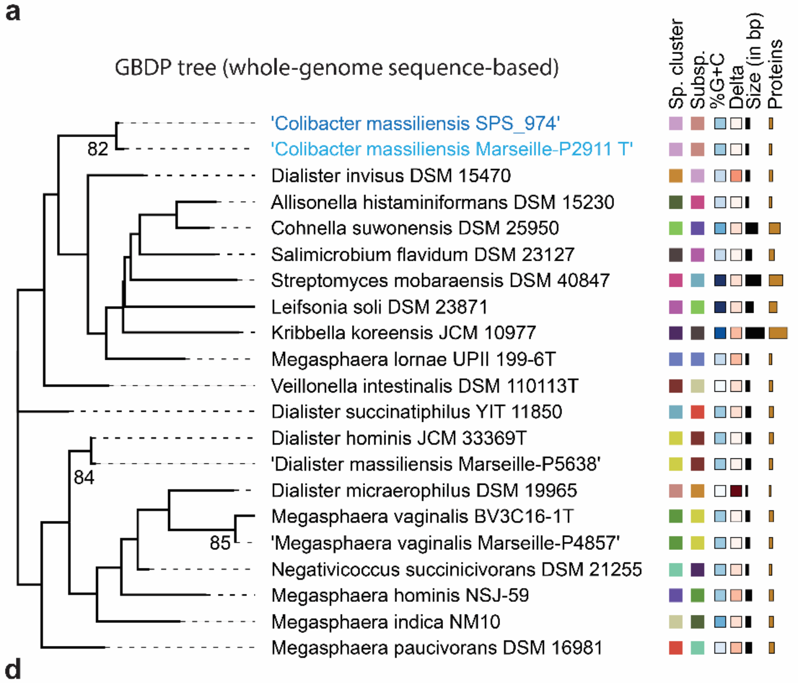

b

Strain	SPS_974	Marseille-P2911
DSM#	n/a	103304
Origin	Oral	Gut
ID	1852379.27	1852379.28
Contigs	1	2
bp	1822736	1715864
Features	1872	1727
Reference	This study	Anani *et al.*, 2019

c

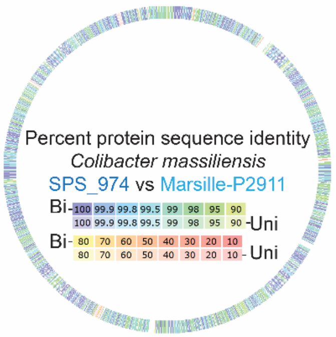

d

Strain	Gene	Role	Category	Subcategory	Subsystem
Marseille	1852379.28.peg.862	CRISPR-associated protein, Csy2 family	DNA Metabolism	CRISPs	CRISPRs
Marseille	1852379.28.peg.861	CRISPR-associated protein, Csy3 family	DNA Metabolism	CRISPs	CRISPRs
Marseille	1852379.28.peg.860	CRISPR-associated protein, Csy4 family	DNA Metabolism	CRISPs	CRISPRs
Marseille	1852379.28.peg.547	Type III restriction-modification system methylation subunit (EC 2.1.1.72)	DNA Metabolism	no subcategory	Restriction-Modification System
Marseille	1852379.28.peg.1409	Adenylosuccinate synthetase (EC 6.3.4.4)	Nucleosides and Nucleotides	Purines	Purine conversions
Marseille	1852379.28.peg.579	programmed frameshift-containing	Protein Metabolism	Protein biosynthesis	Programmed frameshift
Marseille	1852379.28.peg.779	Aspartyl-tRNA(Asn) amidotransferase subunit C (EC 6.3.5.6)	Protein Metabolism	Protein biosynthesis	tRNA aminoacylation, Asp and Asn
Marseille	1852379.28.peg.779	Glutamyl-tRNA(Gln) amidotransferase subunit C (EC 6.3.5.7)	Protein Metabolism	Protein biosynthesis	tRNA aminoacylation, Glu and Gln
Marseille	1852379.28.peg.561	Similarity with glutathionylspermidine synthase (EC 6.3.1.8), group 2	Stress Response	Oxidative stress	Glutathionylspermidine and Trypanothione
SPS_974	1852379.27.peg.252	Cystathionine gamma-lyase (EC 4.4.1.1)	Amino Acids and Derivatives	Lysine, threonine, methionine, and cysteine	Methionine Biosynthesis
SPS_974	1852379.27.peg.365	CRISPR-associated protein Cas2	DNA Metabolism	CRISPs	CRISPRs
SPS_974	1852379.27.peg.369	CRISPR-associated protein, Csd1 family	DNA Metabolism	CRISPs	CRISPRs
SPS_974	1852379.27.peg.1708	Type I restriction-modification system, restriction subunit R (EC 3.1.21.3)	DNA Metabolism	no subcategory	Restriction-Modification System

Supplementary Figure 10. Comparative genomics of two *Colibacter massiliensis* strains from distinct habitats.

a. Whole genome sequence-based comparison between two *Colibacter massiliensis* strains and closely related strains from the TYGS database. Tree inferred with FastME 2.1.6.1 from GBDP distances calculated from genome sequences. The branch lengths are scaled in terms of GBDP distance formula d5. The numbers above branches are GBDP pseudo-bootstrap support values > 60 % from 100 replications, with an average branch support of 20.3 %. The tree was rooted at the midpoint. **b**. Characteristics of two *C. massiliensis* strains. **c**. % protein sequence identity between two strains across genome. Genome of strain SPS_975 was aligned to the genom of strain Marseille and bidirectional or unidirectional best hits were shown as a circular heatmap. **d**. Comparison of metabolic reconstruction of two strains obtained with SEED. The table list all genes which were associated with a subsytem in the respective strain. Source genome data for SPS_974 (this study) are available at

[https://www.ncbi.nlm.nih.gov/bioproject/1297070], and its analysis, including the generation of Figure panel a, can be reproduced using GGDC and associated tools at [https://ggdc.dsmz.de/ggdc.php#]. Reference genome data for Marseille-P2911 were retrieved from [https://www.ncbi.nlm.nih.gov/bioproject/341975]. Panels b–d can be reproduced using both genome sequences in combination with the RAST server at [https://rast.nmpdr.org/].
