# Supplementary material for: Laser-assisted microbial culturomics
Source: Nat Commun. 2025 Nov 26;16:10614. doi: 10.1038/s41467-025-66804-7 (PMC12661035; doi:10.1038/s41467-025-66804-7)
Supplement: Supplementary file 2 — Description of Additional Supplementary Files [file 41467_2025_66804_MOESM2_ESM.pdf]

Description of Additional Supplementary Files

File Name: Supplementary Data 1
Description: List of microbe-microbe interactions supporting growth in co-cultures.
A custom database presents interactions among oral species, in which one species provides vital growth factors to another. Information was retrived from literature. Each row represents a relationship between taxa, detailing the 'helper' taxon and the supported taxon, along with information on culture conditions, additional comments, and references. Species are cross-linked to the eHOMD database.

File Name: Supplementary Data 2
Description: 16S rRNA amplicons sequencing data at the species/OTU level.
For each sample and species/OTU raw read counts are provided.

File Name: Supplementary Data 3
Description: 16S rRNA amplicons sequencing data at the ASV level.
Raw read counts are provided for each sample and ASV, along with the corresponding ASV taxonomy and sequences.

File Name: Supplementary Data 4
Description: Diversity indecies of microbial community structures.
Fifteen community structure metrics were computed on resampled data to assess diversity at the ASV, species, and genus levels. The calculated diversity indices included total feature count (S, also Hill number N0), Margalef's richness index (d), Pielou's evenness (J'), Brillouin's diversity (H), Fisher's alpha, rarefaction at n = 100 [ES(100)], Shannon entropy (H', log base e), Simpson's diversity (1 - λ'), as well as Hill numbers: N1, N2, N∞, N10, N10', N21, and N21'.

File Name: Supplementary Data 5
Description: Identification of species with enzyme assays and based on fluorescence upon excitation with ultraviolet light. Strains with specific activity or characterized by fluorescence were listed.

File Name: Supplementary Data 6
Description: Characteristics of isolates. Partial 16S rRNA gene amplicons sequences are provided. MALDI-based classification is indicated.

File Name: Supplementary Data 7
Description: Most-active peri-implantitis genera in culture collection.
The average relative abundance of the top 50 genera is provided, along with the number of reference strains, reference species, isolated species, and strains in our collection, as well as their presence across four culturomes. All species names are listed.

File Name: Supplementary Data 8
Description: Fastidious species isolated with laser-assisted culturomics.
Fastidious species of ecological or clinical relevance are listed. Inclusion criteria were: novel or unnamed species; species reported to be hard to isolate or identify from a clinical perspective; understudied yet ecologically relevant species with poor genomic references. Brief characteristics

are given for species sorted in alphabetic order. Taxonomic classification is given at species or genus level. In case of the latter, human Microbiome Taxon (HMT) number or information on the most closely related species is given. Clinical origin of isolates is reported; H stands for plaque from a healthy patient while P stands for plaque from a patient with peri-implantitits. Culture conditions and detection method are given. Clinical or ecological relevance of the species are included.

File Name: Supplementary Data 9
Description: Isolation failures: potential cause and proposed solutions.
Taxa that could not be bioprinted or isolated are listed, along with potential reasons for failure and suggested solutions, supported by relevant references

File Name: Supplementary Data 10
Description: Characteristics of sampled individuals.
For each patient collection site, the sampling procedure and additional details are provided, along with information on the sample analysis methods.
