# Supplementary material for: Laser-assisted microbial culturomics
Source: Nat Commun. 2025 Nov 26;16:10614. doi: 10.1038/s41467-025-66804-7 (PMC12661035; doi:10.1038/s41467-025-66804-7)
Supplement: Supplementary file 13 — Reporting Summary [file 41467_2025_66804_MOESM13_ESM.pdf]

nature portfolio

Corresponding author(s): Szymon P. Szafranski
Last updated by author(s): Jul 30, 2025

Reporting Summary

Nature Portfolio wishes to improve the reproducibility of the work that we publish. This form provides structure for consistency and transparency in reporting. For further information on Nature Portfolio policies, see our Editorial Policies and the Editorial Policy Checklist.

Statistics

For all statistical analyses, confirm that the following items are present in the figure legend, table legend, main text, or Methods section.

n/a	Confirmed	
☐	☒	The exact sample size (*n*) for each experimental group/condition, given as a discrete number and unit of measurement
☐	☒	A statement on whether measurements were taken from distinct samples or whether the same sample was measured repeatedly
☐	☒	The statistical test(s) used AND whether they are one- or two-sided<br>*Only common tests should be described solely by name; describe more complex techniques in the Methods section.*
☒	☐	A description of all covariates tested
☐	☒	A description of any assumptions or corrections, such as tests of normality and adjustment for multiple comparisons
☐	☒	A full description of the statistical parameters including central tendency (e.g. means) or other basic estimates (e.g. regression coefficient) AND variation (e.g. standard deviation) or associated estimates of uncertainty (e.g. confidence intervals)
☐	☒	For null hypothesis testing, the test statistic (e.g. *F*, *t*, *r*) with confidence intervals, effect sizes, degrees of freedom and *P* value noted<br>*Give P values as exact values whenever suitable.*
☒	☐	For Bayesian analysis, information on the choice of priors and Markov chain Monte Carlo settings
☒	☐	For hierarchical and complex designs, identification of the appropriate level for tests and full reporting of outcomes
☒	☐	Estimates of effect sizes (e.g. Cohen's *d*, Pearson's *r*), indicating how they were calculated

Our web collection on statistics for biologists contains articles on many of the points above.

Software and code

Policy information about availability of computer code

Data collection	Pacbio sequencing was performed with Pacbio Sequel system; SMRT Link 10.1,
Data analysis	Primer 7, TagCleaner 0.16, UPARSE as implemented in USEARCH 10.0.240, R package dada2 (v3.13), RAST, Type (Strain) Genome Server

For manuscripts utilizing custom algorithms or software that are central to the research but not yet described in published literature, software must be made available to editors and reviewers. We strongly encourage code deposition in a community repository (e.g. GitHub). See the Nature Portfolio guidelines for submitting code & software for further information.

Data

Policy information about availability of data

All manuscripts must include a data availability statement. This statement should provide the following information, where applicable:

- Accession codes, unique identifiers, or web links for publicly available datasets
- A description of any restrictions on data availability
- For clinical datasets or third party data, please ensure that the statement adheres to our policy

The raw 16S rRNA gene amplicon sequencing data is available in GenBank and the European Nucleotide Archive under Project ID PRJEB75613 [https://www.ebi.ac.uk/ena/browser/view/PRJEB75613]. The assembled Colibacter massiliensis genome is available under Accession: PRJNA1297070 [https://www.ncbi.nlm.nih.gov/bioproject/1297070]

Research involving human participants, their data, or biological material

Policy information about studies with human participants or human data. See also policy information about sex, gender (identity/presentation), and sexual orientation and race, ethnicity and racism.

Reporting on sex and gender	n/a
Reporting on race, ethnicity, or other socially relevant groupings	n/a
Population characteristics	The study tested oral biofilms from 15 participants, including 12 healthy volunteers and 3 patients with peri-implantitis.
Recruitment	Healthy volunteers were recruited to provide dental plaque samples for method development. The peri-implantitis cohort forms part of an ongoing clinical cross-sectional study conducted at the Department of Prosthetic Dentistry and Biomedical Materials Science, Hannover Medical School, Germany, within the framework of the 'SIIRI Biofilm Implant Cohort (BIC)'. SIIRI (Safety Integrated and Infection Reactive Implants) is an interdisciplinary research initiative aimed at recruiting hundreds of participants and longitudinally monitoring their peri-implant microbiomes over a period of up to 12 years.
Ethics oversight	The collection of biofilm samples was authorized by the ethics committee of Hannover Medical School (No. 5544 and 9477). Informed consent was obtained from all participants.

Note that full information on the approval of the study protocol must also be provided in the manuscript.

Field-specific reporting

Please select the one below that is the best fit for your research. If you are not sure, read the appropriate sections before making your selection.

☒ Life sciences ☐ Behavioural & social sciences ☐ Ecological, evolutionary & environmental sciences

For a reference copy of the document with all sections, see nature.com/documents/nr-reporting-summary-flat.pdf

Life sciences study design

All studies must disclose on these points even when the disclosure is negative.

Sample size	No sample size estimation was performed. Sample size is similar to one from previous similar study(e.g., doi 10.1038/s41587-023-01674-2)
Data exclusions	Data exclusion was based on sequencing coverage. Single technical replcia from single sample was excluded.
Replication	Hierarchical cluster analysis of microbial 16S rRNA gene fingerprints confirmed the reproducibility of bioprinting.
Randomization	Randomization was not applied.
Blinding	Blinding was not applied

Reporting for specific materials, systems and methods

We require information from authors about some types of materials, experimental systems and methods used in many studies. Here, indicate whether each material, system or method listed is relevant to your study. If you are not sure if a list item applies to your research, read the appropriate section before selecting a response.

Materials & experimental systems

n/a	Involved in the study	
☒	☐	Antibodies
☒	☐	Eukaryotic cell lines
☒	☐	Palaeontology and archaeology
☒	☐	Animals and other organisms
☒	☐	Clinical data
☒	☐	Dual use research of concern
☒	☐	Plants

Methods

n/a	Involved in the study	
☒	☐	ChIP-seq
☒	☐	Flow cytometry
☒	☐	MRI-based neuroimaging

Plants

Seed stocks	*Report on the source of all seed stocks or other plant material used. If applicable, state the seed stock centre and catalogue number. If plant specimens were collected from the field, describe the collection location, date and sampling procedures.*
Novel plant genotypes	*Describe the methods by which all novel plant genotypes were produced. This includes those generated by transgenic approaches, gene editing, chemical/radiation-based mutagenesis and hybridization. For transgenic lines, describe the transformation method, the number of independent lines analyzed and the generation upon which experiments were performed. For gene-edited lines, describe the editor used, the endogenous sequence targeted for editing, the targeting guide RNA sequence (if applicable) and how the editor was applied.*
Authentication	*Describe any authentication procedures for each seed stock used or novel genotype generated. Describe any experiments used to assess the effect of a mutation and, where applicable, how potential secondary effects (e.g. second site T-DNA insertions, mosiacism, off-target gene editing) were examined.*
